# Lower Urinary Tract Neoplasia

**DOI:** 10.3390/vetsci5040096

**Published:** 2018-11-27

**Authors:** Maureen A. Griffin, William T. N. Culp, Robert B. Rebhun

**Affiliations:** School of Veterinary Medicine, University of California-Davis, 1 Garrod Drive, Davis, CA 95616, USA; magriffin@ucdavis.edu (M.A.G.); rbrebhun@ucdavis.edu (R.B.R.)

**Keywords:** neoplasia, urinary, lower urinary tract, bladder, carcinoma, prostate, transitional cell carcinoma

## Abstract

Lower urinary tract neoplasia in companion animals is a debilitating and often life-threatening disease. Tumors of the bladder, urethra, and prostate often occur independently, although extension of these tumors into adjacent regions of the lower urinary tract is documented frequently. The most common lower urinary tract tumor in dogs and cats is transitional cell carcinoma (TCC). In both dogs and cats, TCC affecting the urinary bladder is generally considered to be highly aggressive with both local and metastatic disease potential, and this disease poses unique treatment challenges. Whereas much literature exists regarding the TCC disease process, treatment options, and prognosis in dogs, relatively few studies on feline TCC have been published due to the lower incidence of TCC in this species. Prostate tumors, most commonly adenocarcinomas, occur less commonly in dogs and cats but serve an important role as a comparative model for prostate neoplasia in humans. This article serves as a review of the current information regarding canine and feline lower urinary tract neoplasia as well as the relevance of these diseases with respect to their human counterparts.

## 1. Background

The urinary bladder is the most common site of lower urinary tract neoplasia in both dogs and cats [1,2]. In dogs, the urinary bladder is also the most common site of neoplasia in the entire urinary system (including both upper and lower urinary tracts), and in cats, the urinary bladder is the second most common site of neoplasia in the urinary tract (after renal lymphoma) [1,2]. Urinary bladder neoplasia comprises approximately 2% of all canine malignancies, and the most prevalent urinary bladder cancer in dogs is transitional cell carcinoma (TCC) [3,4,5,6,7,8,9]. Metastatic urinary bladder tumors are infrequently reported in dogs, but local extension from urethral or prostatic tumors into the urinary bladder occurs commonly [5,10]. Bladder neoplasia occurs less frequently in cats, with a reported incidence of 0.07–0.18% [11]. However, a recent multi-institutional, retrospective study included 118 cats with urinary bladder TCC, and the incidence of this disease in cats may be greater than previously suspected [12]. 

Prostate tumors in dogs occur less frequently than urinary bladder tumors with a reported incidence of 0.2–0.6% [13,14,15,16,17]. The most common type of prostatic neoplasia in dogs is adenocarcinoma [15]. Although the incidence is low, the spontaneous development of this cancer in dogs (which is rare in other domestic species) has led to further evaluation of canine prostatic carcinoma as a comparative model for prostatic carcinoma in humans [15,18,19]. TCC of the prostatic urethra frequently invades the prostate, such that it can be difficult to determine whether TCC in the prostate is primarily of prostatic epithelial origin or secondary to invasion from a urethral tumor [18]. Prostatic tumors in cats are very rare, and few published reports of feline prostatic adenocarcinomas exist [20,21,22].

## 2. Transitional Cell Carcinoma of the Lower Urinary Tract in Dogs

### 2.1. Neoplastic Behavior and Classification

In general, TCC is an aggressive, highly invasive tumor with a predisposition for a trigonal location in dogs [3,4,5,6,7]. Most canine lower urinary tract TCCs are reported as high grade, papillary, infiltrative tumors [7,8,9]. In a study of 102 dogs with urinary bladder TCC (the majority of which involved the trigone), TCC also involved the urethra in 56% of dogs and the prostate in 29% of male dogs [5]. Local disease, with frequent trigonal location and urethral involvement, can result in clinical signs associated with lower urinary tract disease (dysuria, hematuria, pollakiuria, and stranguria) and obstruction of the urinary tract [3,8,9]. A guideline for staging of canine bladder neoplasia has been recommended in accordance with the World Health Organization (WHO) in order to guide therapy and provide information on prognosis [23]. Importantly, the bladder tumor grade and TNM stage in accordance with this WHO classification system has been demonstrated to correlate with outcome in these patients [5,7]. According to this criteria, 78% of dogs that are diagnosed with TCC have been reported to have T2 stage disease (tumors that invade the bladder wall), and 20% of dogs with TCC have been reported to have T3 stage disease (tumors that invade adjacent viscera including prostate, uterus, vagina, and pelvic canal) [8,9]. An updated classification scheme for canine TCC was suggested in 2006 [24].

Canine TCC has a high rate of metastasis, with lymph node metastasis in 16%, distant metastasis in 14%, and both nodal and distant metastasis in 10% of dogs at the time of diagnosis [5]. Documented metastasis at the time of diagnosis has been associated with a worse prognosis in these dogs [3,9]. At the time of death, distant metastasis has been reported in 49–58% of dogs with TCC [5,8]. Despite the high incidence of metastatic disease, progression of local disease with subsequent urinary tract obstruction is the cause of death for many dogs with TCC in which the primary tumor is not adequately controlled [5]. Alternatively, in canine patients with adequate primary tumor control, metastatic disease is seen more frequently than urinary tract obstruction at the time of death [5]. This finding demonstrates the importance of both local and systemic treatment in these patients, as the disease is both locally invasive/aggressive and has a high rate of metastasis. In a study of 102 dogs, the cause of death (known for 85/102 dogs) was associated with the primary tumor in 61% of dogs, metastatic disease in 14% of dogs, and non-TCC related problems in 25% of dogs [5]. In a report on 137 dogs with TCC that had necropsies performed, 67% of dogs had documented metastasis in any location with nodal metastasis in 42% of dogs, regional (abdominal, pelvic, and/or inguinal lymph nodes) metastasis in 29% of dogs, any distant metastasis in 58% of dogs, pulmonary metastasis in 50% of dogs, and skeletal metastasis in 11% of dogs [8,9]. 

### 2.2. Risk Factors and Clinical Signs

Risk factors for lower urinary tract TCC in dogs include female sex, historical spay or neuter, breed (particularly Scottish Terriers with a 21-fold increased risk relative to mixed breeds), obesity, exposure to certain flea control products and lawn chemicals, potential exposure to cyclophosphamides, and living in an industrial area [2,4,5,8,9,25]. TCC typically affects older dogs, and the mean age at diagnosis has been reported at 11.05 years [5]. The most common clinical signs associated with TCC in dogs include stranguria, hematuria, and pollakiuria, though other possible clinical signs include tenesmus, lethargy, lameness, and weight loss [2,3,5,9]. Duration of clinical signs ranges from weeks to months prior to diagnosis [3,9].

### 2.3. Diagnosis

Definitive diagnosis of lower urinary tract TCC is vital to providing appropriate prognostic information and treatment recommendations [9]. Definitive diagnosis of TCC typically entails histopathological assessment of tissues obtained via surgery, cystoscopy, or catheter biopsy [3,4,8,26,27]. Transurethral cystoscopic biopsy may be a greater asset in female dogs than male dogs, however, as this method has been reported to accurately diagnose disease in 96% of female dogs but only 65% of male dogs with confirmed lower urinary TCC [28]. Cytology also has a role in diagnosis of TCC, although urine cytology (i.e., assessing for malignant transitional cells in urine) can result in both false negative and false positive results and should be interpreted with caution [9]. Though percutaneous aspirates or biopsies can be adequate means of attaining a diagnosis, these methods may increase the propensity for TCC to seed within the abdomen or body wall [3,4,9,29,30,31]. A TCC urine antigen test has been reported as a sensitive diagnostic test, but false positives are possible [9,32]. A droplet digital PCR assay to detect a BRAF V595E gene mutation has also been described for dogs with TCC [33,34]. This assay has a reported sensitivity of 85% and specificity of 100% when used on free catch urine samples of dogs with both urothelial and prostatic carcinoma [33]. Therefore, a positive BRAF mutation test result can be considered as diagnostic for urothelial (or prostatic) carcinoma in dogs, and this test serves as a valuable minimally invasive tool for obtaining a diagnosis in these patients.

### 2.4. Staging

In dogs with confirmed or suspected lower urinary tract TCC, thorough evaluation of systemic health and staging of the disease should be performed. Evaluation should include physical examination (including rectal exam), complete blood count, serum biochemistry panel, urinalysis, urine culture, thoracic radiographs, abdominal ultrasound, and additional lower urinary tract imaging [3,9]. Findings on rectal examination can include urethral and trigonal thickening or mass effect and sublumbar lymphadenopathy [3,9]. To reduce the risk of neoplastic seeding (though relatively rare), urine may be obtained via voiding or catheterization rather than cystocentesis [3,9]. Because dogs with TCC are predisposed to development of secondary urinary tract infections, and because the clinical signs of lower urinary tract TCC often mimic those of a urinary tract infection, urine culture is recommended [9,35]. Thoracic and abdominal imaging can be valuable staging tools to assess for evidence of metastatic disease in the lungs, lymph nodes, liver, and other locations [9]. If lameness is documented, skeletal radiographs, computed tomography (CT), and/or nuclear scintigraphy may be indicated to assess for possible bone metastasis [9]. Imaging of the urinary tract is important in order to determine tumor location, extent, and feasibility of surgical intervention, evaluate for prognostic information, and obtain baseline measurements to aid in monitoring response to treatment [9]. Imaging modalities for obtaining this information include ultrasound, cystography (which may include double-contrast cystogram, intravenous urogram, and retrograde urethrocystogram), and CT [36,37,38,39].

### 2.5. Treatment

Reported treatments for dogs with lower urinary tract TCC include surgery, radiation therapy, medical therapy (primarily including cyclooxygenase (COX) inhibitors and chemotherapy), and local intravesical therapy. Potential indications for surgical intervention in dogs with TCC include acquisition of a biopsy for diagnosis, excision of the tumor, and restoration or maintenance of urine flow [3,9]. The role of surgical excision in the treatment of dogs with TCC is limited in most cases due to the typical trigonal location, high frequency of urethral involvement, potential for diffuse malignant transformation of the urothelium (“field effect”), and commonly documented metastatic disease at the time of diagnosis [3,5,9]. Techniques for trigonal excision, total/subtotal cystectomy, and incorporation of grafting materials to reconstruct the urinary bladder have been described in dogs, but these techniques are rarely used in clinical patients due to a high risk of morbidity and significant expense [4,5,9,40,41,42,43,44,45]. In a report on 102 dogs with TCC that underwent partial cystectomy or cytoreductive (debulking) surgery, complete resection (i.e., tumor free margins on histopathology) was documented in only 2/102 dogs [5]. In two studies on dogs that underwent partial cystectomy or cytoreductive surgery, median survival times were reported at 106 days and 125 days [2,46]. One possible reason for poor outcomes in dogs that undergo partial cystectomy or a cytoreductive procedure for lower urinary tract TCC involves the potential development of post-operative recurrence or multifocal/diffuse TCC in the urinary bladder; possible causes for this finding include the “field effect,” in which the entire bladder surface undergoes malignant transformation, and/or seeding within the bladder lumen (which may occur secondary to surgical manipulation) [3,47]. An important consideration in dogs that undergo surgical procedures related to lower urinary tract TCC is the potential for peritoneal seeding. One report has documented seeding of TCC to the peritoneal wall in 24/544 dogs, and dogs that underwent cystotomy/cystectomy procedures had an increased incidence of this seeding phenomenon (18/177, 10.2%) compared to dogs that did not undergo cystotomy/cystectomy procedures (6/367, 1.6%) [9,31]. In that report, dogs that were diagnosed with spread of TCC to the peritoneal wall had a poor prognosis with a median survival time of 57 days [31]. These findings emphasize the importance of careful techniques (including both surgical and non-surgical) in an effort to minimize seeding of this neoplasm. 

Due to the historically poor outcomes, the role of surgery in management of dogs with lower urinary tract TCC has become largely palliative in nature with procedures aimed at maintenance or restoration of urinary tract patency without directly altering tumor progression. In particular, placement of urethral and ureteral stents has been described for this purpose, and minimally invasive techniques for these procedures have become available [48,49,50,51]. Urethral stents are now more commonly placed than cystostomy tubes to maintain urinary flow in the event of urethral obstruction in dogs due to a reduction in complications and compliance issues with stents [9,49,50,51]. In two studies, urethral stents were successful in relieving urethral obstruction in 58/61 dogs [49,50]. Possible complications following urethral stent placement procedures include urinary incontinence, stent migration, re-obstruction with continued tumor growth, and increased risk of urinary tract infections [9,49,50]. Median survival time of 78 days has been reported after stent placement, although this number increased to 251 days when adjuvant treatment was also performed (i.e., stent placement performed after nonsteroidal anti-inflammatory drug (NSAID) administration and prior to chemotherapy); moreover, the majority of owners reported satisfaction with the outcome following stent placement [49,50]. Other minimally invasive interventional approaches that have been described to debulk urethral tumors in dogs, and thereby maintain patency of the urethra, include cystoscopic transurethral resection (via electrocautery) and cystoscopic transurethral near-infrared diode laser ablation [52,53]. Transurethral resection of TCC in dogs is somewhat limited due to the invasive nature of tumor into the wall of the lower urinary tract (rather than more superficial, intraluminal forms that can be seen in humans with this disease) [9]. One reported risk of laser ablation is perforation of the urinary tract, and further evaluation of the effect of cystoscopic laser ablation on outcomes in dogs with TCC is needed [9,53].

Radiation therapy has been performed infrequently for treatment of TCC in dogs. In one report on intra-operative radiation therapy (i.e., radiation following partial excision of the tumor with the surgical site exposed) in 11 dogs with urinary bladder TCC, median post-treatment survival time was 15 months and TCC recurrence was documented in 6/11 dogs [54]. Side effects associated with radiation therapy in this study included pollakiuria, urinary incontinence, cystitis, stranguria, and hydronephrosis, and 4/11 dogs were euthanized secondary to such adverse events [54]. Another pilot study documented the use of palliative external beam radiation therapy in conjunction with mitoxantrone and piroxicam administration in 10 dogs [55]. Clinical improvement and stable disease was reported in 7/10 dogs and the median survival time was 240 days; minimal side effects associated with radiation therapy were reported in this study [55]. More recent studies have demonstrated improvement in radiation therapy protocols that enable more accurate treatment of the tumor site and reduced irradiation of normal tissues [56,57]. Additional studies are needed to further investigate the role of radiation therapy as well as optimal protocols for its use in the treatment of canine lower urinary tract TCC. 

In dogs with TCC, systemic medical therapies including chemotherapy agents and COX inhibitors (NSAIDs) are considered to be mainstay treatments [3,8,9]. Multiple chemotherapeutic protocols and drugs have been studied (including cisplatin, carboplatin, mitoxantrone, doxorubicin, vinblastine, and gemcitabine), and in general canine TCC is considered relatively resistant to chemotherapy [3,4,5]. Although long-term outcome data is limited, evidence suggests a potential benefit of chemotherapy administration via an intraarterial route [58]. As compared to canine patients that received intravenous carboplatin for treatment of lower urinary tract carcinoma, patients that received intraarterial carboplatin were more likely to have a tumor response and experienced greater decreases in tumor size [58]. A clinical trial on the use of piroxicam in 76 dogs with TCC demonstrated complete remission in 2.6% of dogs, partial remission in 18.4% of dogs, stable disease in 59.2% of dogs, and progressive disease in 19.7% of dogs; the median survival time of these dogs was 244 days [59]. Prior to this data, the response rate to any single-agent medical therapy protocol (chemotherapeutic drug or NSAID) was less than 25% with a survival time of approximately 6 months or less [1,5]. Based on this study, however, piroxicam may be a reasonable single-agent approach for treatment of lower urinary tract TCC in dogs [9]. Another study demonstrated a potential role for firocoxib as a single-agent therapeutic approach for dogs with TCC; in this study, firocoxib induced partial remission in 20% and stable disease in 30% of dogs with urinary bladder TCC [60]. However, multimodal medical therapy has resulted in an improved survival time in dogs with TCC. For instance, dogs treated with both mitoxantrone and piroxicam had a median survival time of 291 days [61]. The currently accepted and recommended approach to medical therapy in dogs with lower urinary tract TCC involves sequential administration of multiple drugs (chemotherapy agents and NSAIDs) over the course of the disease [3]. The approach outlined by the Purdue University Veterinary Teaching Hospital involves initiation of a medical treatment protocol with monitoring of treatment response every 4-8 weeks and subsequent changes in the protocol based on tumor response and treatment tolerability [3]. Although medical therapy is not considered curative in dogs with lower urinary tract TCC, by following this protocol, TCC growth has been reportedly controlled in approximately 75% of dogs and median survival times have reached 1 year or greater in these canine patients [3,9].

Intravesical therapy has also been reported in dogs with lower urinary tract TCC. A clinical trial documented the administration of intravesical mitomycin C (MMC) in dogs with TCC [62]. Although partial remission and stable disease occurred in 5/13 and 7/13 dogs, respectively, severe systemic side effects (myelosuppression and gastrointestinal signs) occurred in 2/13 dogs [62]. Due to the potential for systemic absorption and adverse effects, intravesical MMC is not recommended at this time [9,62]. Other treatment modalities for canine TCC are emerging, and these include metronomic chemotherapy, folate-targeted therapy, and demethylating agents [9]. Additional studies are needed to further evaluate their role in the treatment of lower urinary tract TCC in dogs. 

### 2.6. Relevance to Human Lower Urinary Tract Transitional Cell Carcinoma

Transitional cell carcinoma of the lower urinary tract in dogs closely resembles high-grade invasive TCC in humans with similar cellular and molecular characteristics, biological behavior, and response to treatment [4,5,8]. Studies on naturally occurring TCC in dogs add to the data that has been acquired in studies on experimentally induced TCC in rodents; the findings obtained from these studies may initiate improvement in the early diagnosis, treatment, and outcomes in humans and dogs with TCC [8]. For instance, studies that investigate the effect of COX inhibitors, targeted therapies, epigenetic based therapy, and metronomic chemotherapy on canine patients with TCC have shown translational promise for studies in human medicine [8]. One aspect of lower urinary tract TCC that appears to be relatively unique to dogs is that of the high incidence of BRAF genetic mutations, as BRAF mutations are a rarely reported feature of TCC in humans [33,63]. However, this information may serve to enhance the knowledge-base and prompt additional studies on the small subset of human patients with BRAF mutation-positive TCC, or alternatively, can be translated into research on other tumor types in humans that are more commonly associated with BRAF mutations, such as malignant melanoma [63].

## 3. Transitional Cell Carcinoma of the Lower Urinary Tract in Cats

Because the incidence of lower urinary tract TCC is much lower (0.07-0.18%) in cats relative to dogs, very few feline TCC studies have been published [11]. Until recently, the largest retrospective study on feline TCC documented 20 cases of urinary bladder TCC in cats [64]. However, a recent multi-institutional, retrospective study reported on 118 cats with lower urinary tract TCC [12]. Treatments performed on these cats included partial cystectomy, NSAIDs, chemotherapy, radiation therapy, and urethral/ureteral stent placement [12]. Tumor location was significantly more variable in these cats than in dogs with TCC, and a trigonal tumor location was reported in 27.1% of the cats [12]. At study completion, metastatic disease was documented in 21.2% of all cats [12]. Primary tumor local progression or recurrence occurred in 55.6% of cats (with recurrence following cystectomy reported in 21.2% of cats post-operatively) [12]. The median survival time for all cats was 155 days, and treatment was significantly associated with survival [12]. The median survival times for untreated cats, cats treated without cystectomy, and cats treated with cystectomy were 46 days, 176 days, and 294 days, respectively [12]. Cats with trigonal disease and a presenting complaint of lethargy were significantly less likely to receive treatment [12]. On multivariable analysis, surgical excision (cystectomy) and NSAID administration were significantly associated with longer survival in cats with lower urinary tract TCC [12].

## 4. Prostatic Carcinoma in Dogs

### 4.1. Neoplastic Behavior and Classification

The majority of prostate tumors in dogs are carcinomas, and of those, most are adenocarcinomas [18]. Prostate carcinoma in dogs generally arise from urothelial or duct cells rather than acinar cells, and most canine prostatic tumors are androgen receptor-negative and, subsequently, androgen independent [18,65,66,67,68,69,70,71]. Moreover, cyclooxygenase-2 (COX-2) expression, though not seen in normal prostate cells, has been detected in 75% of canine prostate carcinoma cells [72]. Historical subclassification of prostatic carcinomas in dogs has been based on differentiation of these tumor cells and include glandular, urothelial, squamoid, and sarcomatoid forms [15]. However, a more recently developed classification scheme is based on growth patterns and includes papillary, cribriform, solid, small acinar/ductal, signet ring, and mucinous tumor types [73]. Regardless of the classification method, prostate carcinoma in dogs appears to be a uniformly aggressive neoplasm. Local invasion and metastasis are common with canine prostatic carcinoma [18]. One study on 76 dogs with prostate carcinoma documented metastatic disease in 80% of the dogs at necropsy [15]. The most common sites of metastasis included lungs and lymph nodes [15]. Prostatic carcinoma in dogs also has a predilection for bone, and skeletal metastasis has been reported in 22–42% of dogs with prostate carcinoma; the most common sites of skeletal metastasis include the lumbar vertebrae and pelvis [15,18,69,74,75]. As with human prostatic carcinoma, this propensity for skeletal metastasis may be associated with both osteolytic and osteoblastic potential of these prostate carcinoma cells [76]. Some evidence suggests that younger dogs with prostate carcinoma have an increased risk of metastasis, although the effect of castration status on this finding is unclear [15,18,77]. Although metastasis is a common component of this disease process, sequellae of the locally aggressive nature of prostate carcinomas, including pain, severe dyschezia, and urinary obstruction, are often the cause of death/euthanasia in these dogs [78].

### 4.2. Risk Factors and Clinical Signs

Prostatic tumors are most commonly seen in older dogs with a mean age of 10 years at diagnosis [14,15]. Although prostate carcinomas have been detected in both intact and castrated male dogs, an increased risk of this disease has been demonstrated in castrated male dogs with an odds ratio of approximately 2.3:4.3 [13,65,68,79,80]. Importantly, this finding is in contrast to the increased risk of development of bacterial prostatitis and benign prostatic hyperplasia in intact male dogs relative to castrated male dogs [81]. Moreover, castrated male dogs may be predisposed to more aggressive prostate tumors with a higher risk of metastasis compared to intact males [18,79]. Benign prostatic hyperplasia has not been documented as a risk factor for development of prostate carcinoma in dogs [82]. Several dog breeds have been reported to have an increased risk of developing prostate carcinoma; those include the Bouvier des Flandres, Doberman Pinscher, Shetland Sheepdog, Scottish Terrier (even when accounting for the increased risk of TCC in this breed), Beagle, Miniature Poodle, German Shorthaired Pointer, Airedale Terrier, and Norwegian Elkhound [68,80]. Common clinical signs in dogs with prostate carcinoma include dysuria, hematuria, stranguria, dyschezia, tenesmus, and flattened/ribbon-like stools (secondary to rectal compression from the prostatic mass and/or metastatic lymph nodes) [15,75,78,79,83]. Patients can present with urinary obstruction secondary to prostatic compression of or tumor extension into the urethra [18]. Other presenting complaints include pain, lameness or gait abnormalities (secondary to tumor invasion of lumbar vertebrae and/or nerve roots), constipation, and non-specific signs of illness such as lethargy, inappetance, and weight loss [18].

### 4.3. Diagnosis

Definitive diagnosis of prostate carcinoma is obtained via biopsy and/or cytology [18,78,83,84]. Means of obtaining samples for cytology include traumatic catheterization, prostatic massage, prostatic wash, ejaculation, ultrasound-guided fine needle aspiration, and impression smears from biopsy samples [18]. Methods for obtaining samples for biopsy include percutaneous, perineal/transrectal, and surgical [18]. Cytology or histopathology can also be performed on suspected metastatic lesions (such as lymph nodes) to aid in diagnosis and staging of this disease in dogs [18]. Similar to TCC of the urinary bladder, there is a risk of seeding prostate carcinoma cells along needle tracts during aspiration or biopsy, such that caution should be taken when performing these diagnostic tests [18,29,85].

### 4.4. Staging

Thorough systemic evaluation and staging should be performed on dogs with prostate carcinoma. Complete systemic evaluation includes physical and rectal examination, complete blood count, serum biochemistry panel, urinalysis, and urine culture. An enlarged, painful, asymmetric prostate is the most common finding on physical and rectal examination, and rectal examination can also reveal enlarged sublumbar lymph nodes [18,85]. Other possible findings on physical examination include musculoskeletal pain and/or lameness and a palpable abdominal mass [85]. Findings on complete blood count and serum biochemistry profile can include anemia, leukocytosis (particularly if secondary infection is present), and hypercalcemia and/or elevated alkaline phosphatase (particularly if skeletal metastasis is present) [18,78]. Urinalysis changes are typically non-specific, though signs consistent with urinary inflammation or secondary urinary tract infection can be detected [18,78]. Thoracic and abdominal imaging should be performed via thoracic radiographs and abdominal ultrasound and/or radiographs. Imaging may also include skeletal survey radiographs and CT or MRI scans. Thoracic radiographs can reveal pulmonary and/or skeletal metastasis [15,74,79]. Abdominal radiographs can reveal prostatomegaly, prostate mineralization, sublumbar or retroperitoneal lymphadenopathy, and periosteal reactions of the vertebrae, pelvis, or femur [18,75,79,86,87]. Abdominal ultrasound can reveal heterogeneous changes of the prostate gland, disruption of the prostatic capsule, sublumbar lymphadenopathy, and evidence of other visceral metastasis [15,79,85,86,87]. Transrectal ultrasound is another recently described modality for obtaining further information about the prostate and locoregional lymph nodes [88]. Although prostatic mineralization in dogs with neoplasia has not been associated with prognosis, the finding of mineralization on imaging can help to guide clinical diagnosis. Mineralization of a prostatic mass is highly sensitive (84%) and specific (100%) for neoplasia in castrated dogs; alternatively, in intact dogs, prostatic mineralization (regardless of the extent of mineralization) may occur with neoplasia, paraprostatic cysts, benign prostatic hyperplasia, or prostatitis [86,89,90]. Survey bone radiographs and/or bone scintigraphy can be performed to help assess for skeletal metastases in patients in which a clinical suspicion for this phenomenon is present [18,85,91]. Skeletal metastases in dogs with prostate carcinoma can consist of osteoblastic, osteolytic, or mixed osteoblastic and osteolytic lesions; when these metastases occur in long bones, they can affect the diaphysis or metaphysis [74,82,92].

### 4.5. Treatment

Multiple treatment modalities have been reported in dogs with prostatic carcinoma. In general, however, the prognosis for these dogs is guarded due to the aggressive nature of this disease (both locally and systemically) and relatively poor response of the disease to conventional treatments [78]. The reported median survival time for dogs without treatment is typically less than 30 days, and many dogs are euthanized at the time of diagnosis [15,93]. A standard-of-care consensus therapy for dogs with prostatic tumors does not currently exist (although administration of NSAIDs is often recommended at a minimum), and therapy has historically largely been considered palliative [18,82]. Although androgen deprivation therapy is a common treatment modality in men with prostate carcinoma, hormonal therapy is unlikely to be effective and is not indicated in dogs with prostate carcinoma due to the typically insignificant role of androgens in canine prostate cancer [15,78,80,82]. Treatment strategies that have been reported in dogs with prostate carcinoma include palliative therapy for maintenance of urethral patency, NSAID administration, chemotherapy, surgery, radiation therapy, palliative therapy for alleviation of pain associated with skeletal metastasis, and minimally invasive interventional procedures including intraarterial chemotherapy, transarterial embolization, and thermal ablation.

In patients with partial or complete urethral obstruction secondary to prostate carcinoma, management strategies may include urethral stent placement, prazosin administration, and cystostomy tube placement [78]. As previously discussed, urethral stents can be placed in a minimally invasive fashion with successful relief of urethral obstruction, and this technique has largely replaced the use of cystostomy tubes in dogs [51,94]. One study on seven dogs with prostatic carcinoma and secondary urinary tract obstruction (partial or complete) demonstrated that administration of prazosin (an alpha-1 antagonist) can be used to temporarily alleviate dysuria and obstruction in these patients [95].

Canine prostatic carcinoma frequently expresses COX-1 and COX-2, and the administration of NSAIDs has resulted in an improved outcome in dogs with prostate tumors [93]. One study on dogs with prostate carcinoma showed that dogs treated with piroxicam or carprofen survived significantly longer (6.9 months) than dogs that were not treated with NSAIDs (0.7 months) [93]. Alternatively, the role of chemotherapy in dogs with prostate carcinoma is unclear, and there exists little evidence to date to support a recommendation of conventional chemotherapy agents in the treatment of canine prostatic carcinoma [18,78]. One study demonstrated effectiveness of nanoparticulate paclitaxel when used in vitro on canine prostate cancer cells, though further studies are needed to determine the effectiveness of this agent in clinical patients [96].

Surgery for canine prostatic carcinoma includes total and partial prostatectomy techniques. Although total prostatectomy can relieve signs associated with urethral obstruction, significant complications (including urinary incontinence) and progression of local and systemic disease have been reported post-operatively without a clear survival benefit in these patients [18,52,82,85,97,98]. However, in a recent study on 25 dogs with prostate neoplasia, total prostatectomy, when combined with adjunctive therapies such as NSAIDs and chemotherapy, resulted in a median survival time of 231 days [99]. Major post-operative complications that required a subsequent surgical procedure occurred in 4/25 dogs that underwent total prostatectomy [99]. Permanent post-operative urinary incontinence was present in 35% (8/23) of dogs with follow-up information after total prostatectomy [99]. Subtotal intracapsular prostatectomy has also been described as an alternative to total prostatectomy, and fewer adverse effects have generally been reported secondary to this procedure compared to total prostatectomy [100]. One prospective study on 21 dogs demonstrated an improved median survival time in dogs that underwent subtotal intracapsular prostatectomy (130 days) relative to dogs that underwent total prostatectomy (17 days) [100]. However, 2/11 dogs that received subtotal intracapsular prostatectomy and 3/10 dogs that received total prostectomy experienced severe post-operative complications and were euthanized within 2 weeks post-operatively [100]. Partial prostatectomy via a neodymium:yttrium-aluminium-garnet (Nd:YAG) laser filleting technique has also been reported in an effort to allow preservation of the prostatic urethra and subsequently reduce the incidence and severity of post-operative urinary incontinence in dogs [18,101,102]. However, a risk of significant post-operative complications still exists with this technique, and one study reported that 3/8 dogs with prostate carcinoma that underwent this treatment died from complications within 16 days of the procedure [102]. When evaluating the use of photodynamic therapy with 5-aminolevulinic acid in combination with partial prostatectomy via Nd:YAG laser, outcomes were relatively poor with rapid progression of disease and a median survival time of 41 days [102]. Transurethral prostate resection with an electrocautery cutting loop has been performed on three dogs (that also received adjunctive treatment) with prostate carcinoma via cystotomy and antegrade cystoscopy [52]. Improvement in clinical signs was noted in all three dogs, but survival times were short (32, 74, and 264 days post-operatively) and reported complications included tumor seeding, urethral perforation, and urinary tract infection [52]. Overall, evidence to date demonstrates a potential role for surgery in palliation of clinical signs with canine prostatic carcinoma, but surgery has typically not been shown to have a significant survival benefit in these patients and can result in substantial post-operative complications [18,82]. Further studies are needed to help guide surgical indications and modalities in dogs with prostate carcinoma.

Radiation therapy has also been performed for treatment of dogs with prostate carcinoma. One study documented the use of intra-operative orthovoltage radiation therapy on dogs with prostate carcinoma and revealed a median survival time of 114 days [103]. A more recent study on intensity-modulated and image-guided radiation therapy on dogs with genitourinary carcinomas showed potential clinical benefit in 90% of dogs and median survival time of 654 days for all dogs in the study, and dogs with prostate carcinoma had a median event-free survival time of 317 days (where events included disease progression, late radiation-associated toxicosis, death, and loss to follow-up) [57]. Radiation therapy may also have a role in palliation of clinical signs associated with the prostate tumor and skeletal metastases [18,78,82]. Other reported treatment modalities for palliation in dogs with osseous metastasis include the use of bisphosphonates (osteoclast inhibitors) and samarium-153-ethylenediamine-tetramethylene phosphonate [104,105,106].

More recently, novel techniques for treatment of prostate carcinoma in dogs, including embolization and ablation, have been described. One preliminary study on the use of transarterial prostatic embolization in dogs with benign prostatic hypertrophy showed significant decrease in prostate size in 4/7 dogs and increase in prostate size characterized by a large cavity within the prostate in 3/7 dogs; complications were not encountered in any of the dogs [107]. An ongoing study at the authors’ institution has documented that all dogs with prostate tumors that have undergone transarterial prostate embolization have had positive outcomes post-procedure with a decrease in tumor size, improvement in clinical signs, and lack of major complications [108]. Previous studies have documented feasibility of percutaneous ultrasound-guided radiofrequency electrocautery ablation, high-intensity focused ultrasound for subtotal ablation, transurethral radiofrequency ablation with a saline electrode, and irreversible electroporation in canine prostate tissue; one report has documented the use of radiofrequency ablation for treatment of prostate carcinoma in a dog [109,110,111,112,113]. Further studies are needed to determine the clinical applicability and outcome of ablation in dogs with prostate carcinoma.

### 4.6. Relevance to Human Prostatic Carcinoma

Although in general, canine prostatic carcinoma is more aggressive and less differentiated than prostatic carcinoma in humans, canine prostatic carcinoma is an ideal animal model for cases of poorly differentiated, androgen-refractory (hormone non-responsive) prostate carcinoma in men [71,82]. Moreover, despite the low incidence of prostate carcinoma in dogs, the dog is one of the few known domestic species that develop spontaneous prostate neoplasia, and as such, dogs represent an important comparative model to enhance understanding of this disease in people [18]. Data from human prostate carcinoma studies can also be used to advance the management of prostate carcinoma in dogs. For instance, a modified version of the Gleason grading scheme (an important tool for prognostic information regarding prostate cancer in men) has recently been used on canine prostate carcinomas with correlation between high scores and more aggressive neoplastic behavior [114].

## 5. Prostatic Carcinoma in Cats

Prostate neoplasia is considered very rare in cats and literature is subsequently lacking. Several case reports exist, with the majority of reported feline prostate tumors being adenocarcinomas in older castrated male cats [20,21,22,115,116,117]. Because of the limited data, no standard-of-care treatment or prognostic information exists in cats with prostate carcinoma [18]. Metastasis is commonly reported in the few cases of cats with prostate carcinoma, and reported sites of metastasis include lungs, lymph nodes, and pancreas. [20,21,22,115,116,117]. Most of the reported cats did not survive beyond 3 months of diagnosis of prostate carcinoma [20,21,22,115,116,117]. However, one case report documented long-term survival with no evidence of local or metastatic disease two years post-operatively in a cat with low-grade prostatic sarcomatoid carcinoma that underwent prostatectomy; another case report documented a 10 month survival time in a cat that underwent prostatectomy and chemotherapy (doxorubicin and cyclophosphamide) for treatment of prostate carcinoma. [21,22]. Further studies are needed to gain more information on prostatic carcinoma in cats and guide treatment recommendations. 

## 6. Miscellaneous Lower Urinary Tract Tumors

Other less common types of bladder neoplasia in dogs include squamous cell carcinoma, adenocarcinoma, undifferentiated carcinoma, rhabdomyosarcoma, hemangiosarcoma, fibroma, and other mesenchymal neoplasms [7,118,119,120,121,122]. Primary extranodal lymphoma of the urinary bladder has also rarely been reported in dogs and needs to be differentiated from multicentric lymphoma with involvement of the urinary bladder [119]. Polypoid cystitis is another rare urinary bladder disease that has been documented in dogs and is characterized by inflammation, epithelial proliferation, and the occurrence of polypoid masses without histopathological evidence of neoplasia; this condition is often associated with urinary tract infections [123]. 

Aside from carcinoma, other reported canine prostatic tumor types include TCC, squamous cell carcinoma, undifferentiated carcinoma, leiomyosarcoma, leiomyoma, hemangiosarcoma, and lymphoma [79,124,125,126,127]. Benign tumors of the prostate are rare and much less common than malignant prostatic tumors [18]. Transitional cell carcinoma and squamous cell carcinoma comprise the majority of urethral tumors in dogs, and urethral smooth muscle tumors have been reported uncommonly [3].

In addition to carcinomas, benign and malignant mesenchymal tumors and lymphoma have also been reported rarely in the urinary bladder of cats [1]. One study reported on the histopathological diagnosis of 20 urinary bladder neoplasms in cats, and findings included angioma, intravenous leiomyoma, adenocarcinoma, squamous cell carcinoma, TCC, leiomyosarcoma, hemangiosarcoma, lymphoma, and embryonal rhabdomyosarcoma; a distinguishing feature of all squamous cell carcinomas and most sarcomas in these cats was their endophytic nature, as opposed to the exophytic nature of all adenocarcinomas and most TCC in these cats [128].
